# Risk factors associated with hepatitis B and C in rural population of Burera district, Rwanda

**DOI:** 10.11604/pamj.2020.35.43.16226

**Published:** 2020-02-12

**Authors:** Patrick Gad Iradukunda, Thierry Habyarimana, Francois Niyongabo Niyonzima, Ange-Yvette Uwitonze, Tharcisse Mpunga

**Affiliations:** 1 Biomedical Laboratory Sciences Department, Faculty of Applied Fundamental Sciences, Institute of Applied Sciences of Ruhengeri, Musanze, Rwanda; 2 Butaro Hospital, Burera, Rwanda

**Keywords:** Hepatitis B virus, hepatitis C virus, Rwanda

## Abstract

**Introduction:**

Several studies have shown that older people have a higher risk of exposure to viral hepatitis B and C than younger people. This study aimed to determine the seroprevalence of hepatitis B and C and their associated factors in people aged 45+ years old in Burera, a rural district of Rwanda.

**Methods:**

A cross sectional study was conducted from July to December 2017 during a mass campaign of hepatitis B (HBV) and hepatitis C (HCV) screening and vaccination of eligible populations against HBV in Burera District. Blood samples were collected and hepatitis B surface antigen (HBsAg) and an antibody against hepatitis C (Anti-HCV) were detected using an Enzyme-Linked Immuno-Sorbent Assay (ELISA). The associated factors were identified using a structured questionnaire and the data was analyzed using SPSS software.

**Results:**

Of the 374 people included in this study, 53.2% were females. The median age was 56 years old with an interquartile range (IQR) of 50 - 63 years old. The prevalence of HBV and HCV infection was 6.4% and 9.4%, respectively, with 0.3% co-infection rate. Age, social economic level, history of blood transfusion, history of never using a condom, as well as a history of injury with a used sharp material were significantly associated with HCV infection.

**Conclusion:**

The study showed a high seroprevalence of both HBV and HCV in Burera’s elderly population aged 45+ years. Several factors associated with HBV and HCV in this study could be prevented through education and improved hygiene.

## Introduction

Historically, hepatitis has been described as a common cause of jaundice in the young population, which can develop into a chronic condition [1]. The hepatitis B virus (HBV) and hepatitis C virus (HCV) were discovered in the late 20^th^ century as the major causes of hepatitis [2,3]. In their chronic state, apart from being associated with cirrhosis and Hepatocellular Carcinoma (HCC) due to the prolonged immune clearance phase, 3.5% of the world’s population (more than 250 million people) are infected with HBV with about one in five people residing in sub-Saharan Africa (SSA) [4-7] suffering from this disease. Three percent (more than 170 million people) of the world population are infected with HCV with the majority also living in developing countries [3-7]. The transmission of HBV and HCV occur when the virus has been exposed to percutaneous or mucosal membrane. According to Wertheim *et al.* [8], HBV is more easily transmitted through sexual contact, breast-feeding and transfer of other body fluids (saliva, semen, vaginal secretions, menstrual blood, and tears) of infected individuals rather than HCV. Other risks for infection include blood transfusion, unsafe use of therapeutic injectable drugs, sharing razors, and tattooing [9]. Health care workers (HCW), intra-venous drug users (IVDU), patients on hemodialysis, blood product users and people located in high prevalence regions have a higher rate of exposure to both viruses [8]. Published studies describing the burden of these viral infections in the Rwandan population were hospital based [10-17]. One of the recent studies conducted at Rwanda Military Hospital (RMH) showed a high prevalence of active HCV among older patients aged 55+ years [16]. However, risk factors associated with the seroprevalence of hepatitis in the urban and rural districts of Rwanda population are not well understood. This study aimed to determine the factors associated with hepatitis B and C among Burera district´s rural population aged 45+ years.

## Methods

After obtaining ethical approval from Butaro Hospital Ethical Committee and INES review board, a cross-sectional study was conducted in Burera district from July to December 2017. The study population was comprised of adults (45+ years old) who were selected among the Burera population that attended a mass campaign for screening for hepatitis B and C. Verbal consent was obtained from 374 participants. Participants were then asked to provide socio-demographic information and the information related to the exposure on the risk factors associated with HCV and HBV infections. We drew 4 ml of blood from each participant through venipuncture using a vacutainer and an ethylene-diamine-tetra-acetic acid (EDTA) anticoagulant tube. Blood was centrifuged (Universal 320 R) at 3000 rpm. The obtained plasma was kept at -20^°^C until the test day. Murex HBsAg (version 3.0) kit, ELx50 Microplate Strip Washer, and ELx800 Absorbance Microplate Reader were used to determine HBsAg for HBV infection confirmation. The cut off value for each plate was calculated following the kit’s insert where 0.05 was added to the mean of the negative control. The HCV was confirmed by the presence of HCV antibody in the participant plasma using the kit of Murex anti-HCV (version 4.0) with a specificity of 99.88%. The cut off value for each plate was calculated by adding 0.6 to the mean of negative control. Both kits were 100% sensitive and the absorbance, which is equal or greater than the cut off value, was considered as positive. Data were organized and entered in SPSS software version 20. Demographic characteristics were presented using frequencies and percentages, Chi-square (χ^2^) test was used to assess the factors associated with HBV and HCV infections. A *P* value of less than 0.05 was considered significant.

## Results

Of the 374 participants, the median age was 56 years, with an Interquartile range (IQR) of 50 to 63 years, with 53.2% being females and 46.8% being males. Most of the participants were married, working as farmers, with little to no education. The participants in this study were distributed equally across all social economic categories (Table 1). However, 35 of 374 participants (9.4% [95% CI: 6.6-12.8%]) had HCV while, 24 of 374 participants (6.4% [95% CI: 4.1-9.4%]) had HBV. The co-infection rate was 0.3% (95% CI: 0.007, 1.5). Fifteen point two percent (57 of 374) reported having a history of sexually transmitted disease. Only 6.4% (24 of 374) reported having received blood transfusion, which has significant association with HCV (p-value of 0.007). Only 4% (15 of 374) had lived with someone infected with either HBV or HCV. Only 0.3% (1 of 374) had received a vaccine against HBV, and none had HIV. As represented in the Table 2, Table 3, people aged between 58 to 68 years were most likely to be HBV positive with 9 of 81 (11.1%) participants in this age group testing positive for HBV. In terms of HCV, high seropositivity was found in the age group of those 68+ years old, with 13 of 66 (18.4%) participants testing HCV positive. Data assessing risk exposure are presented in Table 4, Table 5. Eighty-nine point six percent (335 of 374) of study participants who had never used a condom were significantly found HCV positive (p-value of 0.037). Sixty-one percent (228 of 374) of those who shared personal items, 60% (224 of 374) who had been injured with a used sharp piercing material had a higher likelihood of HCV positivity (p-value of 0.029).

**Table 1:** Socio-demographic and socio-economic characteristics of participants

Variables	N 373	%
**Gender**		
Male	175	46.8
Female	199	53.2
**Age**		
< 48	55	14.7
[48-58]	175	46
[58-69]	81	21.7
68+	66	17.6
**Marital status**		
Single	0	0
Married	268	71.7
Widowed	93	24.9
Divorced	13	3.5
**Level of education**		
None	164	43.9
Some primary	85	22.7
Completed primary	80	21.4
Some secondary	25	6.7
Completed secondary	15	4.0
University or Higher	5	1.3
**Social economic Level**		
Category 1	126	33.8
Category 2	125	33.5
Category 3	122	32.7
**Occupation**		
Farmer	322	86.1
Civil servant	17	4.5
Self-Employee	10	2.7
NGO employee	6	1.6
Other	19	5.1

**Table 2:** Socio-demographic and socio-economic characteristics of HBV seropositive among study participants

Variable	N	HBsAg+ve (%)	P value
**Gender distribution**			
Female	199	11 (5.5)	0.454
Male	175	13 (7.4)	
**Age group (years)**			
[45-48]	55	4 (7.2)	0.123
[48-58]	172	10 (5.8)	
[58-68]	81	9 (11.1)	
68+	66	1 (1.5)	
**Marital status**			
Married	268	2 (15.4)	0.080
Divorced	13	20 (7.5)	
Widower	93	2 (2.2)	
**Social economic category**			
Category 1	126	7 (5.6)	0.867
Category 2	125	9 (7.2)	
Category 3	122	8 (6.6)	
Category 4	0	0	
**Level of education**			
None	164	9 (5.5)	0.419
Some primary	85	3 (3.5)	
Primary	80	7 (8.8)	
Some secondary	25	3 (12)	
Secondary	15	1 (6.7)	
University or +	5	1 (20)	
**Occupation**			
Farmer	322	21 (6.5)	0.744
Civil servant	17	2 (11.8)	
Self-employee	10	0 (0)	
NGO employee	6	0 (0)	
Other	19	1 (5.3)	

Note: no significant variable at 95% confidence interval (P-value > 0.05).

**Table 3:** Socio-demographic and socio-economic characteristics of anti-HCV positive among participant in the study

Characteristics	N	Anti-HCV+ve (%)	P.value
**Gender distribution**			
Female	199	22 (11.1)	0.23
Male	175	13 (7.7)	
**Age group (years)**			
[45-48]	55	2 (3.6)	0.003*
[48-58]	172	10 (5.8)	
[58-68]	81	10 (12.3)	
68+	66	13 (19.7)	
**Marital status**			
Married	268	22 (8.2)	0.129
Divorced	13	0 (0)	
Widower	93	13 (14)	
**Social economic category**			
Category 1	126	21 (16.7)	0.003*
Category 2	125	7 (5.6)	
Category 3	122	7 (5.7)	
Category 4	0	0	
**Level of education**			
None	164	20 (12.2)	0.375
Some primary	85	6 (7.1)	
Primary	80	8 (10)	
Some secondary	25	0	
Secondary	15	1 (6.7)	
University or +	5	0	
**Occupation**			
Farmer	322	29 (9)	0.313
Civil servant	17	1 (5.9)	
Self-Employee	10	0 (0)	
NGO employee	6	1 (16.7)	
Other	19	4 (21.1)	

Note:

* Significant at 95% confidence interval

**Table 4:** Factors associated with hepatitis B surface antigen seroprevalence

Exposure factors	N	HBsAg +ve (%)	P value
**Blood transfusion history**			
Yes	24	4 (16.7)	0.058*
No	350	20 (5.7)	
**Surgical history**			
Yes	58	7 (6.4)	0.056*
No	316	17 (5.4)	
**Multiple site surgeon**			
Yes	5	1 (20)	0.453
No	41	4 (9.8)	
**Tooth extraction**			
Yes	210	14 (6.7)	0.824
No	164	10 (6.1)	
**Hospitalization in last 2 years**			
Yes	62	4 (6.5)	1
No	311	20 (6.4)	
**Medical field exposure**			
Yes	38	2 (5.3)	1
No	335	22 (6.6)	
**HBV Vaccination**			
Yes	1	0	1
No	373	24 (6.4)	
**Had Multiple sex partners**			
Yes	149	10 (6.7)	0.859
No	224	14 (6.2)	
**Had more than 4 sex partners**			
Yes	31	1 (3.2)	0.447
No	342	23 (6.7)	
**History of using condom**			
Yes	39	3 (7.7)	0.728
No	335	21 (6.3)	
**History of sharing personal materials**			
Yes	228	17 (7.5)	0.306
No	146	7 (4.8)	
**History of ear or nose piecing**			
Yes	7	0	1
No	366	24 (6.6)	
**Injury with a used sharp/piercing material**			
Yes	224	16 (7.1)	0.484
No	150	8 (5.3)	
**Living with Hepatitis B or C infected person**			
Yes	15	2 (13.3)	0.249
No	359	22 (6.1)	
**Had at least one STD**			
Yes	57	1 (1.8)	0.148
No	317	23 (7.3)	
**HIV status**			
Positive	14	0	0.61
Negative	252	17 (6.7)	

Note:

* Borderline significant at 95% confidence interval

**Table 5:** Factors associated with hepatitis C virus antibody seroprevalence

Exposure factors	N	Anti-HCV +ve (%)	P value
**Blood transfusion history**			
Yes	24	6 (25)	0.007*
No	350	29 (8.3)	
**Surgical history**			
Yes	58	4 (6.4)	0.627
No	316	31 (9.8)	
**Multiple site surgeon**			
Yes	5	1 (20)	0.379
No	41	3 (7.3)	
**Tooth extract**			
Yes	210	23 (11)	0.231
No	164	12 (7.3)	
**Hospitalization in last 2 years**			
Yes	62	9 (14.5)	0.129
No	311	26 (8.4)	
**Medical field exposure**			
Yes	38	2 (5.3)	0.599
No	335	33 (9.9)	
**Had Multiple sex partners**			
Yes	149	11 (7.4)	0.28
No	224	25 (10.7)	
**Had more than 4 sex partners**			
Yes	31	1 (2.9)	0.338
No	342	34 (9.9)	
**History of using condom**			
Yes	39	0	0.037*
No	335	35 (10.4)	
**History of sharing personal materials**			
Yes	228	24 (10.5)	0.332
No	146	11 (7.5)	
**History of ear or nose piecing**			
Yes	7	1 (14.3)	0.501
No	366	34 (9.3)	
**Injury with a used sharp/piercing material**			
Yes	224	27 (12.1)	0.029*
No	150	8 (5.3)	
**Living with Hepatitis B or C infected person**			
Yes	15	0	0.379
No	359	35 (9.7)	
**Had at least one STD**			
Yes	57	3 (5.3)	0.328
No	317	32 (10.1)	
**HIV status**			
Positive	14	0	0.374
Negative	252	28 (11.1)	

Note:

* Significant at 95% confidence interval

## Discussion

Hepatitis B and C viral infections are hepatocyte specific agents that lead to all 96% of all viral hepatitis-related deaths [2]. Various studies have been conducted to describe and understand the burden of disease and to recommend preventive measures to eradicate infections. In 2015, the HBV and HCV prevalence were between 4.6-8.5% and 0.7-2.4%, respectively in Africa [7]. We found a higher seroprevalence than other studies conducted in Rwanda which might be due to the increased exposure and decreased immunity of the population in the topic. Other studies found the prevalence ranging from 2.4 to 4.5% for HBV and 1.3 to 5.7% for HCV [10,12,14,15,17]. The co-infection rate observed in this study is similar to the coinfection prevalence observed by Umutesi *et al.* [17]. For the biological sex, many studies have found that females have a higher risk for HBV than males [14,17-19]. But in contrast, in our study population, we found that males might have a higher risk for HBV. Like other studies conducted with the aim of determining HCV infections, hepatitis C infection rate is higher in elderly individuals. The observed findings were similar with a previous study of Umutesi *et al.* [17] and was in concordance with other studies that showed a high number of HCV positive people amongst the older population [12,16,18]. HBV and HCV are transmitted after the percutaneous or mucosal exposure to infected blood [20]. While two of the typical HBV risk factors (surgical history and blood transfusion history) had p-values only slightly greater than 0.05, the other risk factors were not found to be remotely significant. The scarcity of information on the vertical transmission mode of this virus which has not been studied in Rwanda, remain a point of interests for further investigations. In addition to age, low socioeconomic status was reported as another risk factor for HCV [21]. Developing countries have a historically high prevalence of HCV, which is echoed by the findings of this study. Moreover, in this study, HCV antibody was detected amongst those who engage in unprotected sex and those who had an accidental injury with a used sharp object which is contradictory to many studies [14,16,18]. HBV has been efficiently linked with unprotected sex [22]. However, evidence shows that the relation between HCV and unsafe sexual intercourse is a rare case which is highly reported in men who have sex with men [23]. Despite that perspective, the contradiction observed in the population of Burera suggests that cohort studies could more effectively assess the association between this behavior and infection rates.

## Conclusion

We found a high seroprevalence of HBV and HCV infections in Burera citizens aged 45+ years. Despite the fact that HBV and HCV have similar transmission modes, a striking difference in the seroprevalence was observed and only HCV had statistically significant association, including age, social economic level, blood transfusion, lifetime of unsafe sex intercourse and injury with a used sharp/piercing material. This study highlighted the absence of HBV vaccination among the targeted population. Through education and improved hygiene, the identified factors can be prevented. A cohort study is needed to determine the prevalence of HBV and HCV in the general population to better understand associated risk factors.

### What is known about this topic

- The prevalence of HCV infection in Rwanda increases with age, however HBV infection varies within age, and both are high among older person;
- There are no known risk factors associated with viral hepatitis B in Rwanda.

### What this study adds

- The prevalence of HBV and HCV is very high in older citizens in Rwanda;
- Blood transfusion, history of never using condom, injury with a used sharp material and social economic level are associated with HCV among older citizens in Rwanda.
